# Decreased Risk of Renal Calculi in Patients Receiving Androgen Deprivation Therapy for Prostate Cancer

**DOI:** 10.3390/ijerph17051762

**Published:** 2020-03-09

**Authors:** Chien-Yu Lin, Jui-Ming Liu, Chun-Te Wu, Ren-Jun Hsu, Wen-Lin Hsu

**Affiliations:** 1 Department of Pediatrics, Hsinchu MacKay Memorial Hospital, Hsinchu City 30071, Taiwan; mmhped.lin@gmail.com; 2 Department of Medicine, MacKay Medical College, New Taipei City 25160, Taiwan; 3 Division of Urology, Department of Surgery, Taoyuan General Hospital, Ministry of Health and Welfare, Taoyuan 330, Taiwan; mento1218@gmail.com; 4 Department of Medicine, National Yang-Ming University, Taipei 112, Taiwan; 5 Graduate Institute of Life Sciences, National Defense Medical Center, Taipei 114, Taiwan; 6 Department of Urology, Chang Gung Memorial Hospital, Keelung 204, Taiwan; wucgmh@gmail.com; 7 Cancer Research Center, Hualien Tzu Chi Hospital, Buddhist Tzu Chi Medical Foundation, Hualien 970, Taiwan; 8 College of medicine, Tzu Chi University, Hualien970, Taiwan; 9 Department of Radiation Oncology, Hualien Tzu Chi Hospital, Buddhist Tzu Chi Medical Foundation, Hualien 970, Taiwan

**Keywords:** prostate cancer, urolithiasis, renal calculi, androgen deprivation therapy, National Health Insurance Research Database

## Abstract

Renal calculi are common, with male predilection and androgen exposure potentially increasing the risk of renal calculi. Systemic effects of androgen deprivation therapy (ADT) have been observed but the influence of ADT on renal calculi in prostate cancer (PCa) patients is not fully understood. We conducted this population-based study to evaluate the impact of ADT on the subsequent risk of renal calculi. We used the National Health Insurance Research Database of Taiwan to analyze the incidences of renal calculi in ADT patients and non-ADT patients from 2001 to 2013. In total, 3309 patients with PCa were selected. After matching with 1:1 propensity-score analysis, 758 ADT patients with 758 matched non-ADT controls were enrolled in the final analysis. Demographic characteristics were analyzed and Cox regression analysis for calculating the hazard ratios (HR) was performed for the subsequent risk of renal calculi. Finally, 186 (186/1516, 12.3%) patients with diagnosed renal calculi were detected. ADT patients had a lower risk of subsequent renal calculi with an adjusted HR of 0.38 (7% vs. 17.5%, 95% confidence interval (CI) 0.28–0.53; *p* < 0.001) in comparison with the non-ADT group. The Kaplan–Meier curve showed significant differences of cumulative incidences of renal calculi. In conclusion, ADT patients had approximately one-third lower risk of subsequent renal calculi. Further studies are warranted to evaluate the clinical significance.

## 1. Introduction

Prostate cancer (PCa) is a major cancer worldwide, presenting a huge disease burden, and it holds the highest incidence of male cancer in the United States, especially in elderly males [1]. In 2017, an estimated 161,360 new cases of PCa were identified in the United States, with 26,730 cases resulting in death. Androgen receptor signaling plays an important role in the pathogenesis of PCa, whereas deprivation of androgen contributes to a mainstream treatment of PCa [2]. An estimated 500,000 patients for PCa underwent androgen deprivation therapy (ADT) in the United States [2]. Multiple systemic effects of androgen were noted and several adverse effects of long-term deprivation of androgen have been reported. Patients with ADT face high risks of metabolic complications, cardiovascular disease, and fractures [3,4,5]. Patients with ADT also show low risk of subsequent development of certain autoimmune diseases [5,6,7]. The complete impacts of ADT on systemic diseases remain incompletely elucidated. 

Urolithiasis is a common disease accounting for approximately 12% of the population with male predilection [8,9,10]. The impairment of quality of life is significant in renal calculi patients, which remains an important health threat [9,11]. The pathophysiology of renal calculi is multifactorial [11,12,13]. Several kinds of renal calculi have been reported, such as calcium oxalate, calcium phosphate, uric acid, staghorn, etc. Low urine volume and increased secretion of calcium, citrate, or uric acid in urine contribute to the formation of renal calculi. Various systemic metabolic factors may affect the gut, renal absorption, and excretion, and alter the risk of renal calculi. Furthermore, patients with renal calculi were found to experience a high risk of cardiovascular events and ischemic strokes [14]. Conversely, a low risk of renal calculi was observed in schizophrenia [15]. Thus, an interaction between systemic disease and renal calculi may exist. 

Compared with women, males experience two to threefold higher incidences of renal calculi, whose pathogenesis may involve androgen [16,17,18,19]. ADT may alter the risk of renal calculi. A significantly higher level of androgen was observed in patients with renal calculi [16,18,19]. From this viewpoint, ADT may decrease the risk of renal calculi. On the other hand, ADT blocks the production of androgen, resulting in increased bone loss, altered urine calcium secretion, and increased risk of renal calculi formation [20]. The complete impact of ADT on renal calculi remains incompletely understood. In a case-control study published in 2017, a higher risk of renal calculi was reported in ADT patients compared with non-ADT individuals (12/41, 29.3% vs. 2/44, 4.5%) [21]. However, the study population was small, and racial differences might have existed. Therefore, we conducted a large-scale study to investigate the association between ADT and renal calculi.

## 2. Materials and Methods 

### 2.1. Data Source and Study Design

This nationwide, population-based large cohort study was conducted using data from the National Health Insurance Research Database (NHIRD) of Taiwan. The NHIRD is the database of the NHI program, which comprises health and medical records, and is a representative of a nationwide medical database with broad coverage. Furthermore, PCa is a major disease, and patients with a diagnosis have to register a catastrophic illness certification and request a waiver for medical payment. The International Classification of Diseases, 9th revision, Clinical Modification (ICD-9-CM) was used for diagnoses in NHIRD. The diagnosis of PCa was validated by the database of catastrophic illness. Our study was conducted after being approved by the Institutional Review Board of the Tri-Service General Hospital (approval number: TSGHIRB NO B-104-21.).

### 2.2. Study Population

**Figure 1:**
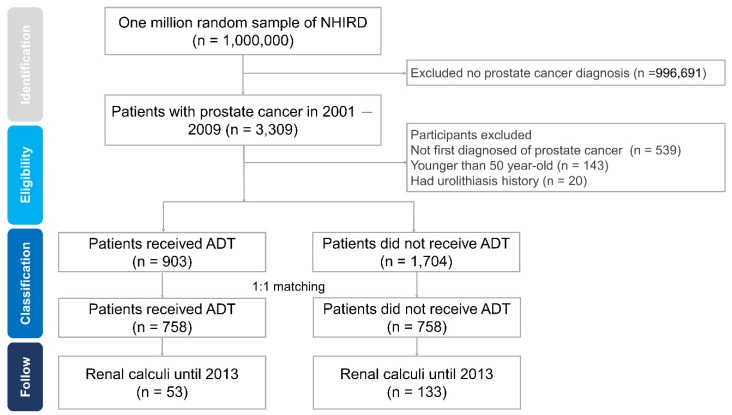
Study flowchart of cohort selection. ADT: androgen deprivation therapy; NHIRD: National Health Insurance Research Database.

Between January 2001 and December 2009, the patients with PCa were identified from the NHIRD data. The diagnoses of PCa were identified by ICD-9-CM codes (ICD-9-CM: 185) [7]. Furthermore, at least 180 days of follow-up time from the initial diagnosis date of PCa was required. Finally, 3309 patients with PCa were identified and considered eligible in this study shown in Figure 1. A total of 903 PCa patients with ADT and 1704 patients without ADT were identified after exclusion. The exclusion criteria were (1) PCa diagnosis before January 1st, 2001 (*n* = 539); (2) patient age younger than 50 years old at the time of diagnosis (*n* = 143); (3) development of renal calculi before the ADT (*n* = 20).

We matched each subject using propensity-score matching analysis with a 1:1 ratio by age group and the selection processes were randomized and blinded. Then, the patients with PCa were classified into four age groups: 50–60, 60–70, 70–80, and >80 years old. We tracked the incidences of renal calculi in ADT and non-ADT groups.

### 2.3. Study Outcomes and Covariates

The use of ADT was verified by drug history and included the use of gonadotropin-releasing hormone agonists (leuprolide, goserelin, triptorelin, and buserelin), oral antiandrogens (cyproterone acetate, bicalutamide, and flutamide), and estrogens (diethylstilbestrol). The outcomes included the incidence of newly diagnosed renal calculi (ICD-9-CM: 592). The diagnoses of renal calculi were mainly conducted by a urologist according to patient history, physical examination, and image findings. All the patients were followed up with for 4 years to explore the incidence of renal calculi. Censoring in this study was characterized as the date of diagnosis of renal calculi, death, or until the end of the 31st December 2013, whichever came first.

Covariates included age at diagnosis, income group, geographic area, urbanization levels. Comorbid diseases were analyzed for both groups; these diseases included hypertension (ICD-9-CM: 401, 402, 403, 404, and 405), hyperlipidemia (ICD-9-CM: 272), diabetes (ICD-9-CM: 250), coronary artery disease (ICD-9-CM: 410, 411, 412, 413, and 414), chronic obstructive pulmonary disease (COPD, ICD-9-CM: 491, 492, and 496), cerebrovascular disease (CVA, ICD-9-CM: 430, 431, 432, 433, 434, 435, 436, 437, and 438), obesity (ICD-9-CM: 278), alcoholism (ICD-9-CM: 291, 303, 5711, 5712, 5713, 7903, A215, V113, 30,500, 30,501, 30,502, and 30,503), and smoking (ICD-9-CM: 496, 3051, 4910, 4912, 4928, 5236, 6490, 98,984, and V1582).

### 2.4. Statistical Analysis

The characteristics of selected patients were analyzed by descriptive statistical method. The χ2 test was used for the comparison of categorical variables. The incidences of renal calculi in both groups were investigated, and Kaplan–Meier (KM) curve was for estimation of the cumulative incidences. The log-rank test was for estimation of the difference between the ADT and non-ADT patients. Hazard ratios (HRs) were used to estimate the association between covariates and renal calculi in propensity score–matched and multivariable-adjusted proportional hazards models. We used software to perform analyses with the SPSS, Version 22.0 for Windows (IBM, Armonk, NY, USA), SAS, Version 9.2 (SAS Institute, Cary, NC, USA) and performed the KM curve with the STATA version 11.2 (StataCorp, College Station, TX, USA). *p* < 0.05 was defined as statistically significant in this study.

## 3. Results

A total of 3309 patients with PCa were identified from NHIRD between 2001 and 2009. Of these PCa patients, 2607 were enrolled after considering the exclusion criteria, that is, 903 subjects with ADT and 1703 without ADT (Figure 1). After propensity score matching, 758 individuals were identified in ADT groups and non-ADT groups with a 1:1 ratio. Table 1 shows the demographic characteristics of the full cohorts. The ADT patients showed higher COPD and tobacco use. No differences were observed in age, income, and other comorbidities. 

During the follow-up period, 186 (12.3%) patients were newly diagnosed with renal calculi; 53 (7.0%) in the ADT group and 133 (17.5%) in the non-ADT patients (Table 2). Compared with non-ADT patients, Cox proportional analysis revealed that the crude HR was 0.37 (95% confidence interval (CI): 0.27–0.52, *p* < 0.001) for incidences of renal calculi in the ADT group. After adjusting for age, comorbidities, and other variables, the adjusted HR of newly diagnosed renal calculi was 0.38 (95% CI: 0.28–0.53; *p* < 0.001) for ADT use (Table 3). Diabetes and CVA were found to be associated with significantly lower risk of renal calculi (adjusted HR = 0.64, 95% CI: 0.44–0.95 and *p* < 0.05 for diabetes; adjusted HR = 0.67, 95% CI: 0.45–0.99, and *p* < 0.05 for CVA).

**Figure 2:**
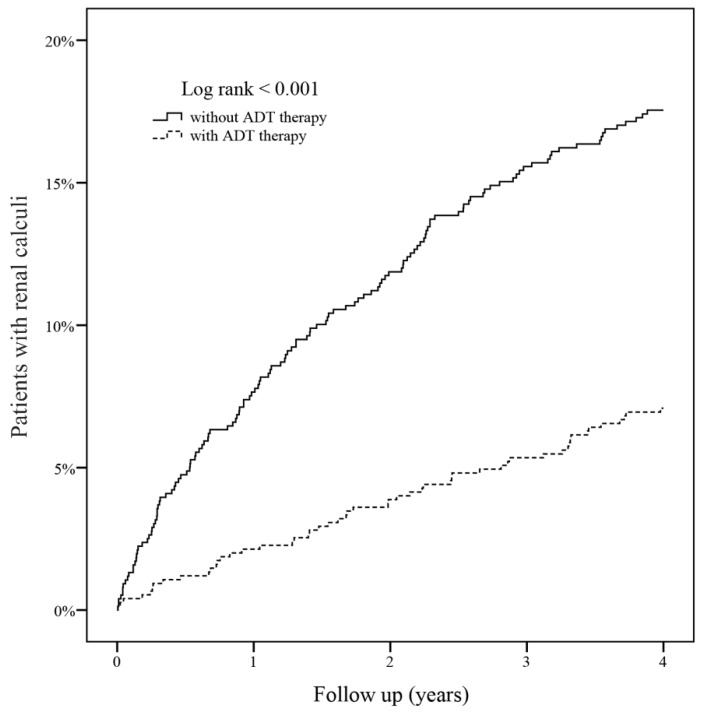
Kaplan–Meier curves according to androgen deprivation therapy use for the incidence of renal calculi in the propensity score–matched cohort. ADT, androgen deprivation therapy.

The KM curve was plotted to compare the incidences of renal calculi, and it showed a significantly lower risk of renal calculi in the ADT group (Figure 2; log-rank test, *p* < 0.001). 

## 4. Discussion

This large-scale cohort study showed a significantly decreased risk of subsequent renal calculi in ADT patients. A total of 1516 patients with PCa were tracked, and patients under ADT showed a lower risk of renal calculi compared with the non-ADT patients. The possible protective effect against renal calculi formation of ADT was demonstrated, but further studies are warranted to investigate the association and mechanisms.

The observed reduction of risk may result from the blockage of androgen. Renal calculi are more prevalent in males and postmenopausal females, indicating the possible role of androgen in renal calculi formation. A significantly higher level of testosterone was reported in those with renal calculi [16,18,19,22]. In Mohammad’s study, the testosterone level was 3.3 ng/mL in patients with renal calculi and 2.41 ng/mL in the control group [18]. Testosterone increases the hepatic glycolic acid oxidase, which is an enzyme responsible for the metabolic pathway of oxalate synthesis and leads to high urinary oxalate secretion [17]. Decreased liver oxalate synthesis and urinary oxalate excretion were observed in androgen receptor knock-out mice. Androgen receptor signaling plays an important role in the pathophysiology of renal calculi formation. However, the underpinning mechanism is complicated and not fully elucidated. Testosterone may also increase the alpha-enolase expression on the surface of renal tubular cells, which involves a crystal cell adhesion process [23]. Therefore, androgen/testosterone may increase the risk of renal calculi via several different mechanisms. Conversely, osteoporosis is an important issue in patients with ADT [24]. ADT will increase bone absorption and urine calcium secretion; an association has been observed between hypercalciuria and renal calculi formation [25,26]. The conflicting effects of ADT on renal calculi caught our attention, and our study showed that the overall impact of ADT will decrease the risk of subsequent renal calculi. Further studies are required to elucidate the underpinning mechanisms.

A case-control study in Spain comparing the risk of renal calculi in ADT and non-ADT patients [21] showed an increased risk of renal calculi in ADT patients (12/41, 29.3% vs. 2/44, 4.5%), and the conclusion is contrary to that of the present study. The age distribution differed in ADT and non-ADT groups (73.6 ± 9.2 vs. 67.1 ± 7.7 years, *p* = 0.001), and the case numbers were relatively small. The risk of renal calculi might be affected by ages and our study had been propensity score–matched for ages. Additionally, the observed risk may change in different countries, races, and patients with different dietary habits. Further studies are required to draw conclusions between ADT and renal calculi. 

Owing to changes in lifestyle and dietary habits, the prevalence of hypertension, diabetes, obesity, hyperlipidemia, and cardiovascular disease have increased worldwide. The formation of urolithiasis is multifactorial and may be affected by various systemic diseases. A high risk of renal calculi was observed in patients with metabolic syndrome [27]. Previous reports revealed a 1.2–1.6-fold risk of renal calculi in patients with type 2 diabetes [27,28,29]. Several mechanisms may contribute to the observed association [29,30,31]. First, insulin resistance may impair renal ammoniagenesis and increase the production of acidic urine and lower urine pH. Second, the resorption of sodium and uric acid in the proximal tubule may be increased by insulin and cause hyperuricemia. Finally, hyperuricemia and hyperuricosuria increase the formation of uric acid stones. However, the complete association was not fully established. Our study revealed a decreased risk of renal calculi in patients with diabetes, but the components of renal calculi were not investigated in the analysis. The proportion of uric acid stone was unavailable, thus requiring further studies. Similarly, controversial, bidirectional effects between hypertension and renal calculi have been reported, and an increased risk of CVA has been observed in previous studies [14,31]. The present study showed no significant influence of hypertension but caused a decreased risk of renal calculi in patients with CVA. The complexity of metabolic syndrome and complex interactions between these diseases causes difficulty in the investigation of the independent effects of single diseases. 

The strength of our study is a large-scale population-based study with enrolment of more than 3000 patients with PCa. However, there were still several limitations. First, data of the laboratory tests were unavailable in the NHIRD, and the components of renal calculi were not analyzed. Although the majority of renal calculi consisted of calcium oxalate and calcium phosphate component in most reports, we were unaware whether ADT shows a different risk with varying kinds of stones. In addition, further investigation of laboratory data could not be estimated. The levels of sex hormones, blood and urine calcium, uric acid, and other laboratory markers were disregarded. Moreover, the definite influences of androgen deprivation status on renal calculi were difficult to identify in this study. The complicated underpinning pathophysiology between ADT and renal calculi cannot be evaluated in the present study. Thus, further prospective studies are warranted to clarify the complete mechanism and association between ADT and renal calculi.

## 5. Conclusions

This nationwide, large-scale, population-based study showed that ADT use in patients with PCa results in approximately one-third lower risk of subsequent renal calculi. This result could give useful information for physicians in managing the complicated risks and benefits of patients with ADT. Further studies are required to gain deeper insights into the relationship between ADT and renal calculi.

## Figures and Tables

**Table 1:** Demographic and medical characteristics of prostate cancer patients according to androgen deprivation therapy.

Characteristics	ADT Patients, *n* (%)	Non-ADT Patients, *n* (%)	*p* Value
No. of cases	758	758	
Gender			
Male	758 (100.0%)	758 (100.0%)	
Age			0.994
50–59	33 (4.4%)	35 (4.6%)	
60–69	143 (18.9%)	141 (18.6%)	
70–79	352 (46.4%)	351 (46.3%)	
≥80	230 (30.3%)	231 (30.5%)	
Insured region			<0.001 *
Northern Taiwan	395 (52.1%)	470 (62.0%)	
Middle Taiwan	110 (14.5%)	101 (13.3%)	
Southern Taiwan	218 (28.8%)	171 (22.6%)	
Other (Eastern Taiwan	35 (4.6%)	16 (2.1%)	
and outlying islands)			
Urbanization			<0.05 *
1 (highest)	341 (45.0%)	393 (51.8%)	
2	151 (19.9%)	161 (21.2%)	
3	170 (22.4%)	122 (16.1%)	
4 (lowest)	96 (12.7%)	82 (10.9%)	
Insured amount NTD ^a^			0.681
<20,000	679 (89.6%)	669 (88.3%)	
20,000–39,999	30 (4.0%)	39 (5.1%)	
40,000–59,999	24 (3.2%)	22 (2.9%)	
≥60,000	25 (3.2%)	28 (3.7%)	
Comorbidity			
Diabetes Mellitus	232 (30.6%)	211 (27.8%)	0.236
Hypertension	477 (62.9%)	443 (58.4%)	0.074
Hyperlipidemia	187 (24.7%)	194 (25.6%)	0.679
Coronary heart disease	278 (36.7%)	273 (36%)	0.789
Cerebral vascular accident	219 (28.9%)	196 (25.9%)	0.185
COPD	279 (36.8%)	227 (29.9%)	<0.05 *
Alcoholism	4 (0.5%)	3 (0.4%)	0.705
Obesity	2 (0.3%)	4 (0.5%)	0.413
Tobacco use disorder	245 (32.3%)	201 (26.5%)	<0.05 *

^a^ NTD refers to New Taiwan dollars, of which 1 U.S. dollar = 30 TWD; * *p* < 0.05; ADT, androgen deprivation therapy; COPD, chronic obstructive pulmonary disease.

**Table 2:** ADT patients and non-ADT patients as predictors of renal calculi by Cox regression analysis.

	Number of Patients	
	ADT Patients *n* = 758	Non-ADT Patients *n* = 758
Renal calculi	53 (7.0%)	133 (17.5%)
Without renal calculi	705 (93.0%)	625 (82.5%)
Crude HR	0.37 (0.27 to 0.52) **	

** *p* < 0.001 for comparison between ADT patients and non-ADT patients; ADT, androgen deprivation therapy; HR, hazard ratio.

**Table 3:** Independent predictors of renal calculi analyzed by Cox regression analysis.

	Crude HR (95% CI)	Adjusted HR (95% CI)
Prostate cancer		
non-ADT	1	1
ADT	0.37 (0.27 to 0.52) **	0.38 (0.28 to 0.53) **
Age		
50–59	1	1
60–69	1.84 (0.84 to 4.04)	1.96 (0.87 to 4.39)
70–79	1.14 (0.53 to 2.46)	1.18 (0.52 to 2.67)
≥80	0.87 (0.39 to 1.93)	0.81 (0.35 to 1.89)
Insured region		
Northern Taiwan	1	1
Middle Taiwan	1.18 (0.79 to 1.76)	1.17 (0.77 to 1.77)
Southern Taiwan	0.72 (0.50 to 1.05)	0.80 (0.53 to 1.19)
Other (Eastern Taiwan	1.03 (0.48 to 2.21)	1.56 (0.70 to 3.46)
and outlying islands)		
Urbanization		
1 (highest)	1	1
2	0.87 (0.59 to 1.27)	0.85 (0.58 to 1.26)
3	0.95 (0.65 to 1.39)	1.10 (0.74 to 1.65)
4 (lowest)	0.71 (0.43 to 1.20)	0.81 (0.47 to 1.43)
Insured amount NTD ^a^		
<20,000	1	1
20,000–39,999	0.80 (0.38 to 1.71)	0.53 (0.24 to 1.15)
40,000–59,999	1.26 (0.59 to 2.67)	0.94 (0.42 to 2.08)
≥60,000	0.75 (0.31 to 1.82)	0.54 (0.22 to 1.34)
Comorbidity disease		
Diabetes Mellitus	0.56 (0.39 to 0.80) *	0.64 (0.44 to 0.95) *
Hypertension	0.72 (0.54 to 0.96) *	1.02 (0.73 to 1.41)
Hyperlipidemia	0.71 (0.49 to 1.01)	0.76 (0.51 to 1.11)
Coronary heart disease	0.78 (0.57 to 1.07)	1.04 (0.74 to 1.46)
Cerebral vascular accident	0.56 (0.39 to 0.82) *	0.67 (0.45 to 0.99) *
COPD	0.55 (0.39 to 0.78) *	0.93 (0.52 to 1.65)
Alcoholism	0.05 (0 to 469.73)	NA
Obesity	0.05 (0 to 977.5)	NA
Tobacco use disorder	0.51 (0.35 to 0.74) **	0.66 (0.36 to 1.23)

^a^ NTD refers to New Taiwan dollars, of which 1 U.S. dollar = 30 TWD; * *p* < 0.05, ** *p* < 0.001; ADT, androgen deprivation therapy; HR, hazard ratio; COPD, chronic obstructive pulmonary disease; CI, confidence interval; NA, not applicable.
